# Hiwi Promotes the Proliferation of Colorectal Cancer Cells via Upregulating Global DNA Methylation

**DOI:** 10.1155/2015/383056

**Published:** 2015-08-18

**Authors:** Lin Yang, Lei Bi, Qingwei Liu, Meng Zhao, Bin Cao, Dong Li, Jianjun Xiu

**Affiliations:** ^1^Department of Radiology, Provincial Hospital Affiliated to Shandong University, Jinan 250021, China; ^2^Department of Radiology, Affiliated Hospital of Qingdao University Medical College, Qingdao 266003, China; ^3^Department of Gastroenterology, Affiliated Hospital of Qingdao University Medical College, Qingdao 266003, China; ^4^Department of Radiology, Tai'an Tumor Hospital, Taian 271000, China

## Abstract

Hiwi is well known for its role in stem cell renewal, maintaining the resting stage, and downregulating cell cycle of stem cells via RNA silencing. And Hiwi overexpression has been recognized in several types of cancers. In the present study, we examined the Hiwi expression in colorectal cancer (CRC) specimens in both mRNA and protein levels via real-time quantitative PCR, western blot assay, and immunohistochemical staining. Then we explored the role of Hiwi in the cancer cell proliferation and in the DNA methylation in human CRC Caro-2 and HT-29 cell lines. Results demonstrated that both mRNA and protein levels of Hiwi were significantly higher in 38 CRC tissues than in 38 peritumor tissues. Moreover, the Hiwi overexpression with an adenovirus vector significantly promoted the proliferation of Caro-2 and HT-29 cells, associated with significant increase in the global DNA methylation levels. And the chemical inhibition of DNA methylation significantly restrained such proliferation promotion. In summary, we confirmed that Hiwi was overexpressed in CRC tissues and that the forced Hiwi overexpression promoted the proliferation and global DNA methylation of CRC cell lines. Our results imply for the first time that Hiwi promotes the proliferation of CRC cells via promoting global DNA methylation.

## 1. Introduction

Colorectal cancer (CRC) is the third most common cancer with an annual incidence of one million cases and an annual mortality of 655,000 individuals, leading to a high incidence of cancer-related death worldwide [1–3]. There is a higher rate of CRCs in developed countries or areas such as USA, Canada, Australia, and Europe [4], and an increasing CRC incidence happens in Asia [4]. However, little is known about the etiology and pathogenesis of CRCs. Epidemiological studies indicate a familial aggregation of CRCs [5, 6]. Furthermore, studies have proved that* Hiwi* gene has the prognostic value for patients with CRC and may play a pivotal role in the tumor development [7, 8] and may be a potential target for cancer therapy.

Hiwi is one of human homologues of P-element induced wimpy testis in* Drosophila* (Piwi) family members, which are characterized by the presence of Piwi and Piwi-Argonaute-Zwille domains, exerting a well-known role in RNA silencing [9]. Hiwi plays a key role in regulation of stem cell renewal, maintaining the resting stage and in downregulating cell cycle of stem cells [10]. Additionally, Hiwi was overexpressed in several types of cancers, such as human adenocarcinomas of the pancreas, gastric cancers, lung cancers, and CRCs [10–12]. Recently, accumulating studies reveal that the Hiwi expression in human cancer cells is associated with proliferation of cancer cells [9, 13, 14]. Additionally, it has been confirmed that the Hiwi expression correlates with DNA methylation level, as the Hiwi downregulation decreases DNA methylation and limits tumorigenic growth [15]. DNA methylation is a primary epigenetic modification regulating gene expression and chromatin structure in eukaryotes [16] and is associated with many biological events. For instance, it was shown that the CpG island hypermethylation was a biomarker for the early detection of lung cancer [17]. However, it is not clear whether the overexpressed Hiwi promotes DNA methylation and thus promotes the proliferation of CRCs.

In this study, to investigate the role of Hiwi in the CRC, we examined the expression of Hiwi in CRC specimens, and then we overexpressed Hiwi in CRC cells to explore the regulation of Hiwi in the proliferation of CRC cells as well as the DNA methylation in CRC cells. Our results demonstrated that the Hiwi overexpression in human CRC cells promoted the DNA methylation and the growth of CRC cells.

## 2. Materials and Methods

### 2.1. CRC Specimens, Cell Culture, and Reagents

In this study, 38 CRC specimens and 38 peritumor specimens (as control; at a distance more than 10 mm from the tumor edge) were obtained from CRC patients with complete clinicopathologic data of specimens recorded. Detailed clinicopathologic data was shown in Table 1. And human CRC specimens in our study were allowed by patients for scientific research and were approved by the institutional ethics committee of our hospital. CRC cell lines, Caco-2 and HT-29, were originally obtained from Shanghai Bioleaf biotech Co. Ltd. (Shanghai, China) and were maintained, respectively, with Eagle's Minimum Essential Medium (EMEM) (Invitrogen, Carlsbad, CA, USA) and McCoy's 5a medium (Sigma-Aldrich, St. Louis, MO, USA) containing 10% fetal bovine serum (FBS; GIBCO, Rockville, MD, USA), penicillin, and streptomycin. The cells were cultured at 37°C in a humidified atmosphere of 5% CO_2_ conditions. The inhibitor of DNA methylation, 5-Aza-2′-deoxycytidine (DAC), was purchased from Sigma-Aldrich (St. Louis, MO, USA) and was dissolved in acetic acid: water solution (1 : 1) for a concentration of 200 *μ*M.

### 2.2. Immunohistochemical Staining for Hiwi

The Hiwi level in 15 of 38 CRC specimens and in 15 of 38 peritumor specimens was firstly immunostained. Tissue slides were firstly deparaffinized and rehydrated serially. Then the antigen was retrieved via heating for 20 min at 98°C in 10-mM sodium citrate (pH 6.0), and the endogenous peroxidase activity was inactivated with 0.4% H_2_O_2_ for 20 minutes. Slides were incubated overnight at 4°C with the primary antibody against Hiwi (1 : 500, ab135003, Abcam, Cambridge, UK) and then with the secondary antibody (Sinabio, Beijing, China) for 30 min at room temperature. Tissue sections were counterstained with Mayer's hematoxylin. The blind evaluation of staining was independently performed by two pathologists. The standard score for staining intensity and area was scored, respectively, as 0 (no staining), 1 (weak staining), 2 (moderate staining), or 3 (intense staining) and as 0 (0%), 1 (1%–25%), 2 (26%–50%), 3 (51%–75%), or 4 (76%–100%) according to the percentage of positively stained cells. The final staining score for each sample was calculated by the sum of the intensity and area scores.

### 2.3. Overexpression of Hiwi with Adenovirus Vector

Human* Hiwi* coding sequence (BC031060) was amplified from the cDNA sequence of Hiwi (Sinobio, Beijing, China) and was cloned into the pShuttle-CMV (Strategene, La Jolla, CA, USA) to generate the recombinant pShuttle-CMV-Hiwi. The replication-deficient adenovirus Ad-HIWI was constructed according to the technical protocol of the AdEasy Vector System (Stategene, La Jolla, CA, USA). In brief, pShuttle-CMV-Hiwi was linearized and was cotransferred into BJ5183 bacterial cells with pAdeasy-1 (the viral DNA plasmid) to generate the recombinant adenovirus plasmid pAdeno-Hiwi by homologous recombination. The pAdeno-Hiwi was then linearized and was transfected into the HEK293 cell line to generate the adenovirus Ad-HIWI. The Red Fluorescent Protein (RFP) was used as a negative control, and the Ad-RFP was generated accordingly. To overexpress Hiwi or RFP in CRC cells, the Ad-HIWI or the Ad-RFP virus with 1 or 10 multiplicities of infection (MOI) was inoculated in the CRC cells for 1 hour in 5% CO_2_ incubators at 37°C. Then the cells were washed with warm (37°C) phosphate buffered saline (PBS) for three times and were updated with fresh EMEM or McCoy's 5a medium supplemented with 2% FBS.

### 2.4. RNA Isolation and RT-qPCR

Total cellular RNA isolation from the intratumor, peritumor specimens or the CRC cells was performed with the Rneasy plus mini kit (Qiagen, Valencia, CA, USA), according to the manufacturer's instructions, and reverse transcription-PCR (RT-PCR) was performed with the Qiagen One Step RT-PCT kit (Qiagen, Valencia, CA, USA). The primers for* Hiwi* were synthesized according to the reported sequences [18]. The mRNA level of Hiwi level was presented as a relative value to GAPDH with the 2^(−Delta  Delta  C(T))^ method [19].

### 2.5. Protein Isolation and Western Blot Analysis

CRC specimens for western blot analysis were collected and lysed with standard RIPA buffer (Boston Bio Products, Boston, USA), according to the manual, and were centrifuged at 10000 g at 4°C for 30 min. Protein samples were separated by 10% SDS-PAGE gel. Nitrocellulose membranes (Bio-Rad, Hercules, CA, USA) were blocked with 5% nonfat dry milk at 4°C overnight and incubated with anti-Hiwi monoclonal antibody for 1 hour at room temperature. The Hiwi band on the membrane was detected after using the secondary antibody (Santa Cruz Biotechnology) and the enhanced chemiluminescence (ECL) detection system (Amersham, Uppsala, Sweden) following the manufacturer's instructions.

### 2.6. Global Methylation Analysis

To analyze the promotion by Hiwi overexpression to the DNA methylation in CRC cells, we examined the global DNA methylation in Caco-2 or HT-29 cells, after the infection with 0, 1, or 10 MOI Ad-Hiwi or Ad-RFP viruses for 24 hours, or (and) after the treatment with 0, 50, 200, 800, or 2000 Mm DAC for 24 hours. Sodium bisulfate conversion of genomic DNA in each sample was performed as described [20]. The DNA methylation was analyzed using MethyLight method [21], which relies on methylation-specific primers and the methylation-specific fluorescent probe. This combination of methylation-specific detection principles results in a highly methylation-specific detection technology, with an accompanying ability to sensitively detect very low frequencies of hypermethylated sites.

### 2.7. Cell Proliferation Assay

CCK-8 and colony assay were used to evaluate cell proliferation according to the manufacturer's instruction. Briefly, Caco-2 or HT-29 cells were post-incubated at 37°C, harvested, and trypsined. 48 hours after infection, cells were seeded into 12-well plates and incubated in DMEM containing 0.5% FBS. The medium was replaced every 24 hours, and 10 mL CCK-8 reagent (DOJINDO, Kumamoto, Japan) was added to each well. Absorbance was read at 450 nm in each well. For the colony formation assay, 500 cells from each group were added to 12-well plate for an incubation of 3–6 days, and the cell colonies were stained by 0.5% crystal violet (Sigma-Aldrich, St. Louis, MO, USA) and were counted directly on the plate.

### 2.8. Statistical Analysis

Statistical analysis was performed by unpaired *t*-tests, after a confirmation of the normal distribution of data from the two group, with Chi-square test, using SPSS16.0 software (IBM SPSS, Armonk, NY, USA). Data are presented as mean ± SD error of the mean (SEM). *p* value < 0.05 (^*∗*^), <0.01 (^*∗∗*^), or <0.001 (^*∗∗∗*^) indicated a statistically significant difference, respectively.

## 3. Results 

### 3.1. Overexpression of Hiwi in CRC Specimens

**Figure 1:**
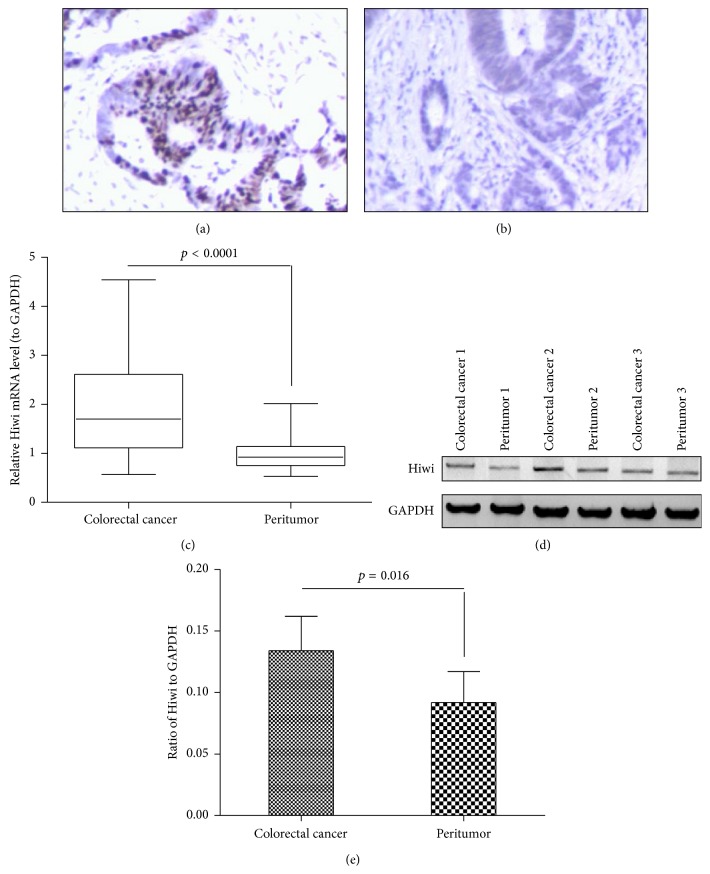
Overexpression of Hiwi protein in CRC specimens. (a) and (b): immunohistochemical staining for Hiwi expression in CRC tissues (*n* = 38) and peritumor specimens (*n* = 38); (c): relative mRNA level of Hiwi in the CRC specimens or the colorectal noncancer specimens by quantitative real-time RT-PCR; (d): Hiwi overexpression at protein level in the CRC or noncolon specimens, revealed by the western blot analysis; (e): percentage of Hiwi to GAPDH in protein level. The *p* value was indicated accordingly.

The expression of Hiwi was firstly evaluated by immunohistochemical staining in 15 CRC specimens and 15 peritumor colorectal specimens. The representative staining for Hiwi in CRC specimens (Figure 1(a)) and peritumor specimens (Figure 1(b)) was indicated. The average staining score for CRC specimens was 2.50 ± 0.67, whereas it was 1.33 ± 0.50 for peritumor specimens (*p* < 0.05). Then all 38 CRC specimens and 38 colorectal peritumor colorectal specimens were examined for Hiwi expression in both mRNA and protein levels by quantitative real-time RT-PCR and western blot analysis. There was a significantly high level of Hiwi mRNA expression in the CRC specimens, compared to the expression in noncancer colorectal specimens (Figure 1(c), 2.009 ± 0.198 versus 1.005 ± 0.07768, *p* < 0.001, paired-samples *t*-test). Then the Hiwi protein level in cells was analyzed by western blot analysis, as shown in Figures 1(d) and 1(e), and the difference at Hiwi protein level was also significant between CRC specimens and noncancer specimens (Figure 1(e), *p* = 0.016). These findings indicated that Hiwi expression is closely associated with the CRC.

### 3.2. Hiwi Overexpressed by Adenovirus Vector in Caco-2 Cells

**Figure 2:**
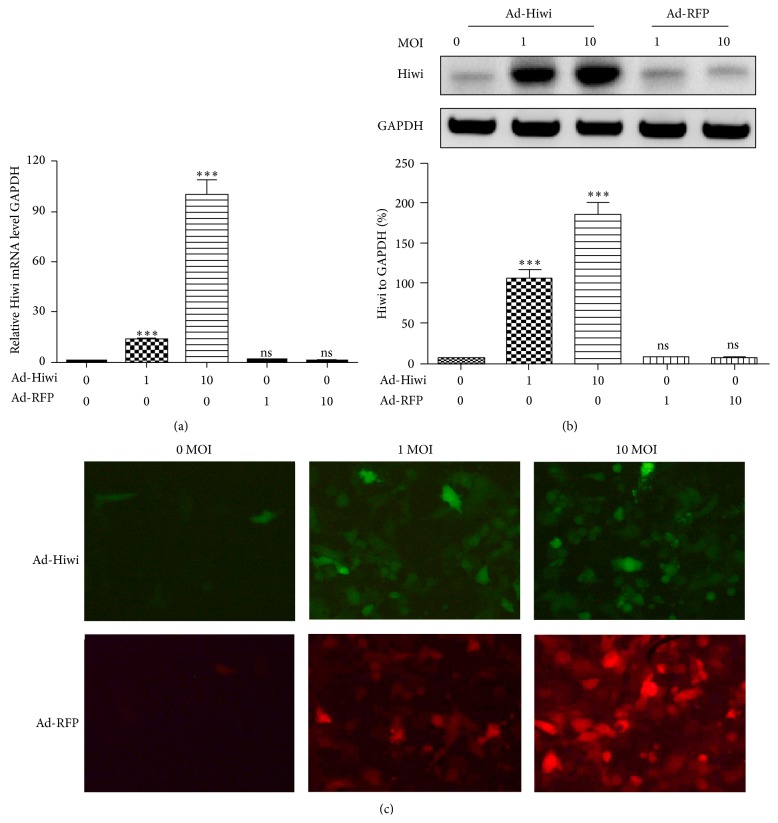
Overexpression of Hiwi by adenovirus vector at a series of MOI. (a) and (b): the expression of Hiwi at mRNA (a) or protein (b) level in the Caco-2 cells, which were infected with 0, 1, or 10 MOI Ad-Hiwi or Ad-RFP. The protein level of Hiwi was expressed as the percent level to GAPDH (b). (c) The expression of Hiwi in the Caco-2 cells, which were infected with 0, 1, or 10 MOI Ad-Hiwi by immunohistochemistry. (d) The RFP fluorescence microscopy of Caco-2 cells infected by Ad-RFP at 0, 1, or 10 MOI. Statistical significance was shown as ^*∗*^ *p* < 0.05, ^*∗∗*^ *p* < 0.01, and ^*∗∗∗*^ *p* < 0.001; ns, no significance.

To investigate the role of Hiwi overexpression in CRC cells, we used an adenovirus vector carrying Hiwi gene to overexpress the Hiwi in Caco-2 or HT-29 cells. Whole coding sequence of Hiwi was cloned into adenovirus vector, and the recombinant adenovirus harboring Hiwi coding sequence (named as Ad-Hiwi) was rescued and determined. Red fluorescence protein gene (RFP) was also cloned into adenovirus vector as control (Ad-RFP). As shown in Figure 2(a), the Hiwi mRNA level in the Ad-Hiwi-infected Caco-2 cells was significantly higher than in the Ad-RFP-infected Caco-2 cells at 1 or 10 MOI. The protein level was also significantly higher in the Caco-2 cells which were infected with the Ad-Hiwi virus than in those infected with the Ad-RFP virus, by western blot analysis (Figure 2(b)). In addition, the immunohistochemistry analysis also indicated a high Hiwi expression in Caco-2 cells which were infected with 1 or 10 MOI Ad-Hiwi; there was a higher level of green fluorescence in the Ad-Hiwi-infected Caco-2 cells (Figure 2(c)), whereas the Ad-RFP infection caused a higher level of red fluorescence in Caco-2 cells at 1 or 10 MOI (Figure 2(c)).

### 3.3. Hiwi Overexpression Promotes the Growth of HT-29 or Caco-2 Cells

**Figure 3:**
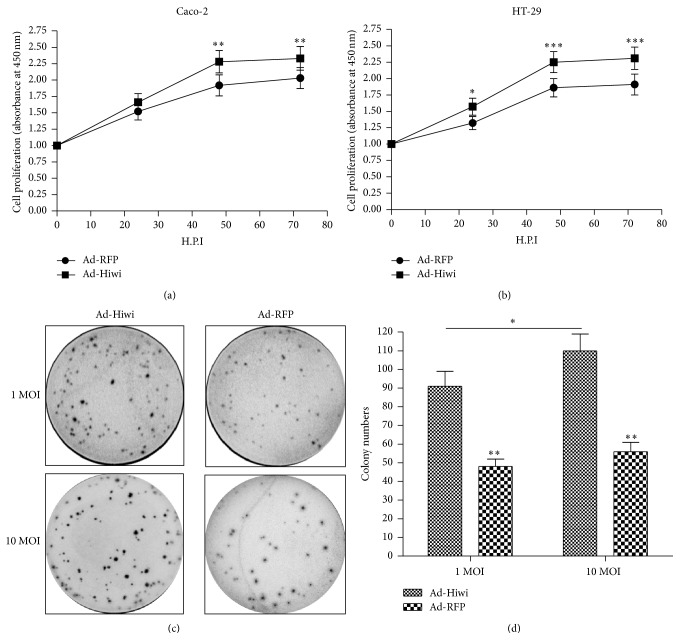
Overexpression of Hiwi promotes the growth of HT-29 or Caco-2 cells. (a) and (b): significantly promoted growth of Caco-2 cells (a) or HT-29 cells (b), which were infected with 1 MOI Ad-Hiwi, with 1 MOI Ad-RFP infection as control. The growth of Caco-2 or HT-29 cells which were infected with Ad-Hiwi or with Ad-RFP was evaluated by CCK-8 assay. (c) and (d): difference in cell growth by colony formation assay between the Ad-Hiwi- and Ad-RFP-infected Caco-2 cells at 1 or 10 MOI. Statistical significance was shown as ^*∗*^ *p* < 0.05, ^*∗∗*^ *p* < 0.01, and ^*∗∗∗*^ *p* < 0.001.

Aiming to evaluate the influence of Hiwi overexpression on the growth of CRC cells, we used the CCK-8 assay and colony assay for this analysis. CCK-8 assay was used for cell counting every 24 hours for Caco-2 or HT-29 cells after the infection with Ad-Hiwi or Ad-RFP, as shown in Figure 3(a); the proliferation rate of Caco-2 cells which were infected with Ad-Hiwi was significantly higher than cells which were infected with Ad-RFP (*p* < 0.01 for 24 or 48 hours postinoculation (H.P.I.)) and the promotion by Ad-Hiwi infection was also confirmed in HT-29 cells (Figure 3(b); *p* < 0.05 or *p* < 0.001 for 24, 48, or 72 H.P.I.). Furthermore, the cell growth difference was determined by colony assay between the Ad-Hiwi- and Ad-RFP-infected Caco-2 cells. It was shown in Figure 3(c) that there were more colonies formed by the Ad-Hiwi-infected Caco-2 cells than the Ad-RFP-infected Caco-2 cells at 1 or 10 MOI (Figure 3(d); *p* < 0.05 for 1 or 10 MOI). Thus, we confirmed the promotion effect of overexpressed Hiwi to the growth of CRC cells by CCK-8 assay and colony forming assay.

### 3.4. Detection of Global Genomic Methylation in Caco-2 or HT-29 Cells after Ad-Hiwi or Ad-RFP Infection

**Figure 4:**
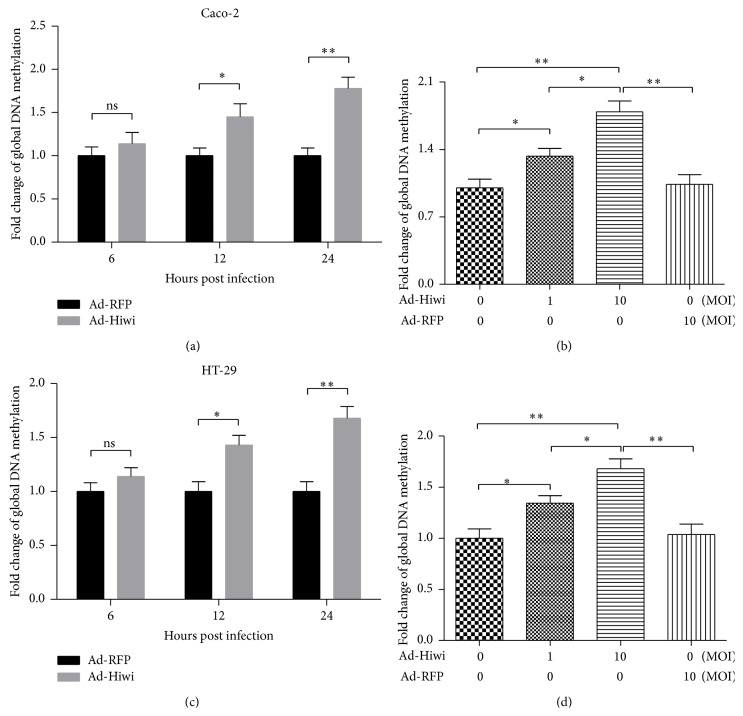
Global genomic methylation level in Caco-2 or HT-29 cells post-Ad-Hiwi or Ad-RFP infection. (a) and (c): time-dependence DNA methylation levels in Caco-2 cells (a) or HT-29 (c) cells, after being infected with Ad-Hiwi or Ad-RFP viruses. (b) and (d): dose-dependence of the methylation promotion by the Ad-Hiwi infection (0, 1, or 10 MOI) in Caco-2 cells (b) or HT-29 (d) cells, with the infection with 10 MOI Ad-RFP as control.

To investigate the association of the growth promotion by Hiwi overexpression with the DNA methylation in CRC cells, we then evaluated the global DNA methylation in the Ad-Hiwi- or Ad-RFP-infected CRC cells. The global DNA methylation fold change in Caco-2 or HT-29 cells after Ad-Hiwi or Ad-RFP infection is shown in Figure 4. There was significant difference of the DNA methylation level between the Ad-Hiwi- and Ad-RFP-infected Caco-2 (Figures 4(a) and 4(b)) or between the Ad-Hiwi- and Ad-RFP-infected HT-29 cells (Figures 4(c) and 4(d)). There was no difference between the blank and Ad-RFP infected Caco-2 or HT-29 cells. And what is more, there was a dose dependence in the DNA methylation promotion by the Ad-Hiwi infection. The fold change of global DNA methylation in Caco-2 or in HT-29 cells was higher after the infection with Ad-Hiwi at 10 MOI than at 1 MOI (Figures 4(b) and 4(d); *p* < 0.05 resp.). These findings showed that DNA methylation level in CRC cells was significantly associated with the Hiwi overexpression.

### 3.5. DNA Methylation Blockage Inhibits the Proliferation Promotion by Ad-Hiwi to Caco-2 or HT-29 Cells

**Figure 5:**
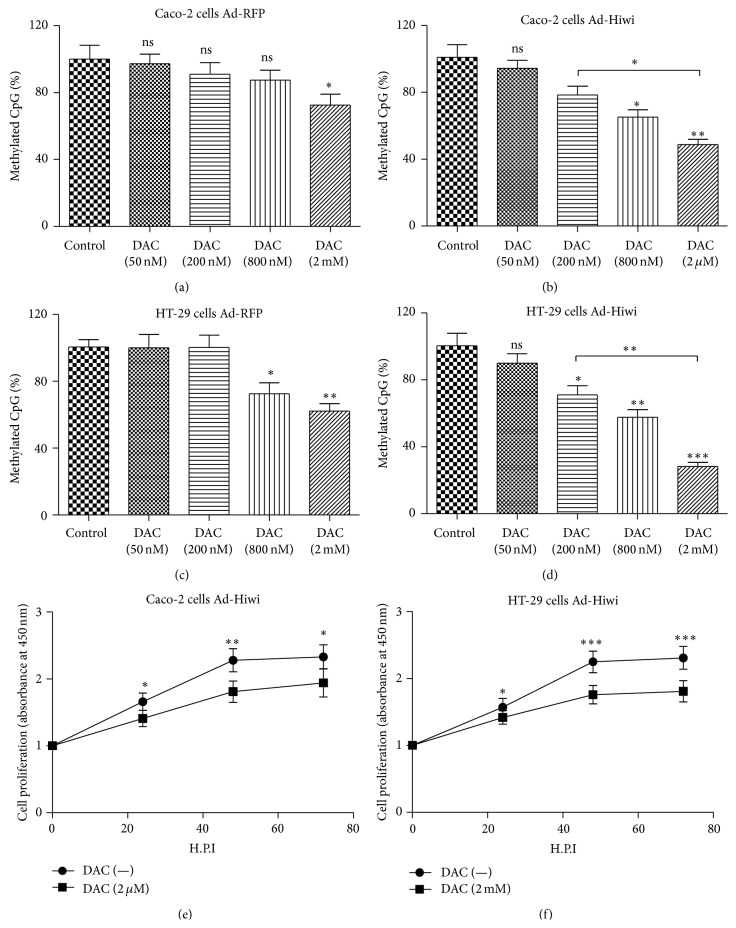
Inhibition of methylation blocked proliferation effect of Ad-Hiwi to Caco-2 or HT-29 cells. (a)–(d): DNA methylation levels in CACO-2 cells ((a), (b)) or HT-29 cells ((c), (d)) infected with 1 MOI Ad-RFP ((a), (c)) or with 1 MOI Ad-Hiwi ((b), (d)) after the treatment with 0, 0.05, 0.2, 0.8, or 2 *μ*M DAC for 24 hours, respectively. (e) and (f): 2 *μ*M DAC inhibited the Ad-Hiwi-promoted proliferation of Caco-2 or HT-29 cells by CCK-8 assay. Statistical significance was shown as ^*∗*^ *p* < 0.05, ^*∗∗*^ *p* < 0.01, and ^*∗∗∗*^ *p* < 0.001; ns, no significance.

Aiming to confirm the influence by DNA methylation on the growth of CRC cells, 5-aza-2′-deoxycytidine (DAC) was used for blocking the DNA methylation in Caco-2 or HT-29 cells which were infected with Ad-Hiwi or Ad-RFP. As shown in Figure 5(a), there was no difference in the DNA methylation level between 0 mM DAC (control) and 0.05, 0.2, or 0.8 mM DAC in Caco-2 cells infected by Ad-RFP; only the DAC of 2 mM significantly reduced the DNA methylation level in the Ad-RFP-infected Caco-2 cells (Figure 4(a); *p* < 0.05). However, the DAC with more than 200 nM significantly reduced the DNA methylation level in the Ad-Hiwi-infected Caco-2 cells (Figure 4(b); *p* < 0.05), dose dependently (*p* < 0.05 for 200 Nm and 2 Mm DAC). Compared to Caco-2 cells infected by Ad-RFP or Ad-Hiwi and without DAC treatment, there was a difference when DAC was more than 0.8 mM in HT-29 cells infected by Ad-RFP and when DAC was more than 0.2 mM in HT-29 cells after the infection with Ad-Hiwi (Figures 5(c) and 5(d)). Moreover, the cell proliferation was determined by CCK-8 assay after the Ad-Hiwi infection and the treatment with 0, 0.05, 0.2, 0.8, or 2 *μ*M DAC for 24 hours. As shown in Figure 5(e), the 2 mM DAC significantly inhibited the promoted cell proliferation by the Ad-Hiwi infection (*p* < 0.05 for 24 or 72 H.P.I., *p* < 0.01 for 48 H.P.I.). And such inhibition by DAC was reconfirmed in HT-29 cells. The proliferation curve of the Ad-Hiwi-infected HT-29 cells treated with 2 mM DAC was also lower than the Ad-Hiwi-infected HT-29 cells without DAC treatment (Figure 5(f)). These findings indicated that DNA methylation knockdown blocked the proliferation promotion by the Hiwi overexpression in CRC cells.

## 4. Discussion

Piwi is first discovered in* Drosophila* germ line stem cells in 1997 and is responsible for stem cell renewal [22]. It interacts with RNAs via the RNA-interference mechanism and plays a role in the proliferation of germinal stem cells [23, 24]. The Hiwi overexpression has been described primarily in hematopoietic stem cells (HSC) and germ cells. In addition, it is involved in various tumors and may play a pivotal role in tumor cell proliferation [7, 9, 18, 25]. Recently, the recognition of the role of Hiwi in tumorigenesis has been updated, Hiwi is shown to be directly tumorigenic, and Hiwi-expressing cancers may be addicted to Hiwi expression. The oncogenic role of Hiwi has recently been focused on. Hiwi overexpression has been confirmed in lung cancers [26], gastric cancers [13], hepatocellular carcinoma [27], cervical cancers [28], and also CRC [8, 29]. And the overexpressed Hiwi is associated with the proliferation and migration of human hepatocellular carcinoma cells [9, 18], with the chemoresistance [28], and correlates with poor prognosis [7, 8, 11, 18, 30]. In the present study, we confirmed the Hiwi overexpression in CRC specimens in both mRNA and protein levels. There was a significant high level Hiwi in CRC specimens than in peritumor specimens. And the adenovirus harboring Hiwi coding sequence significantly promoted the Hiwi level in CRC cells. Moreover, the Hiwi overexpression significantly promoted the proliferation of CRC Caco-2 or HT-29 cells, as was confirmed by the results of both CCK8 assay and colony forming assay.

The Hiwi-associated DNA methylation is reversible along with Hiwi-induced tumorigenesis, and the Hiwi-associated global DNA-hypermethylation occurs in nonpromoter CpG regions [15]. In a mouse model, Piwi orthologs have been more extensively studied in terms of DNA methylation [31, 32]. It was indicated that Hiwi translationally upregulated DNA methyltransferases (DNMTs) and directly upregulated the global DNA methylation (at both CpG and non-CpG sites) [15]. The inducible downregulation of Hiwi in human sarcomas inhibited growth and reestablished differentiation of tumor cells [15]. However, the exact role of the Hiwi-mediated DNA methylation in cancers is still unclear. We found in this study that the Hiwi overexpression in CRC cells significantly upregulated the DNA methylation. And the inhibitor of DNA methylation, DAC, could significantly downregulate the Hiwi-induced DNA methylation and further blocked the Hiwi-promoted proliferation of Caco-2 or HT-29 cells. Interestingly, the methylation reduction caused by DAC was more significant in HT-29 cells than in Caco-2 cells. It seemed that HT-29 cells were more sensitive than Caco-2 cells to the methylation inhibitor, with unknown mechanism.

In conclusion, we confirmed the Hiwi overexpression in CRC tissues. The Hiwi overexpression promoted the proliferation and global DNA methylation of CRC Caro-2 or HT-29 cells, whereas the chemical inhibition of DNA methylation blocked such proliferation promotion. Our results imply for the first time that Hiwi promotes the proliferation of CRC cells via promoting global DNA methylation.

## Figures and Tables

**Table 1:** Characteristics of colorectal cancer patients.

Characteristics	Colorectal cancer (*n* = 38)
Gender	
Male	22
Female	16
Age (range, years)	34–61
Tumor stage^*∗*^	
I	8
IIA	13
IIIA	4
IIIB	11
IIIC	2
Tumor grade	
G1	5
G2	23
G3	10

^*∗*^According to the guidelines for staging colon cancer from National Comprehensive Cancer Network (NCCN): ^*∗*^stage I: T1-T2N0M0; stage IIA: T3N0M0; stage IIIA: T1-T2N1/1cM0 or T1N2aM0; stage IIIB: T3-4aN1/1cM0, T2-T3N2aM0, and T1-T2N2bM0; stage IIIC: T4aN2aM0, T3-T4aN2bM0, and T4bN1-T2M0.

## References

[1] Weng W., Feng J., Qin H., Ma Y. (2015). Molecular therapy of colorectal cancer: progress and future directions. *International Journal of Cancer*.

[2] Abbruzzese C., Diodoro M. G., Sperduti I. (2015). Detection of phosphorylated insulin receptor in colorectal adenoma and adenocarcinoma: Implications for prognosis and clinical outcome. *Journal of Cellular Physiology*.

[3] Balog C. I. A., Stavenhagen K., Fung W. L. J. (2012). N-glycosylation of colorectal cancer tissues: a liquid chromatography and mass spectrometry-based investigation. *Molecular and Cellular Proteomics*.

[4] Ng S. C., Wong S. H. (2013). Colorectal cancer screening in Asia. *British Medical Bulletin*.

[5] Le Marchand L., Zhao L. P., Quiaoit F., Wilkens L. R., Kolonel L. N. (1996). Family history and risk of colorectal cancer in the multiethnic population of Hawaii. *American Journal of Epidemiology*.

[6] Kim Y.-I. (1999). Folate and carcinogenesis: evidence, mechanisms, and implications. *Journal of Nutritional Biochemistry*.

[7] Zeng Y., Qu L.-K., Meng L. (2011). HIWI expression profile in cancer cells and its prognostic value for patients with colorectal cancer. *Chinese Medical Journal*.

[8] Raeisossadati R., Abbaszadegan M. R., Moghbeli M., Tavassoli A., Kihara A. H., Forghanifard M. M. (2014). Aberrant expression of DPPA2 and HIWI genes in colorectal cancer and their impacts on poor prognosis. *Tumor Biology*.

[9] Xie Y., Yang Y., Ji D., Zhang D., Yao X., Zhang X. (2014). Hiwi downregulation, mediated by shRNA, reduces the proliferation and migration of human hepatocellular carcinoma cells. *Molecular Medicine Reports*.

[10] Sharma A. K., Nelson M. C., Brandt J. E. (2001). Human CD34^+^ stem cells express the *hiwi* gene, a human homologue of the *Drosophila* gene *piwi*. *Blood*.

[11] Sun G., Wang Y., Sun L. (2011). Clinical significance of Hiwi gene expression in gliomas. *Brain Research*.

[12] Grochola L. F., Greither T., Taubert H. (2008). The stem cell-associated Hiwi gene in human adenocarcinoma of the pancreas: expression and risk of tumour-related death. *British Journal of Cancer*.

[13] Liu X., Sun Y., Guo J. (2006). Expression of hiwi gene in human gastric cancer was associated with proliferation of cancer cells. *International Journal of Cancer*.

[14] Wang D.-W., Wang Z.-H., Wang L.-L., Song Y., Zhang G.-Z. (2014). Overexpression of hiwi promotes growth of human breast cancer cells. *Asian Pacific Journal of Cancer Prevention*.

[15] Siddiqi S., Terry M., Matushansky I. (2012). Hiwi mediated tumorigenesis is associated with DNA hypermethylation. *PLoS ONE*.

[16] Lee J., Jang H., Shin H., Choi W. L., Mok Y. G., Huh J. H. (2015). AP endonucleases process 5-methylcytosine excision intermediates during active DNA demethylation in *Arabidopsis*. *Nucleic Acids Research*.

[17] Kim Y., Kim D.-H. (2015). CpG island hypermethylation as a biomarker for the early detection of lung cancer. *Methods in Molecular Biology*.

[18] Zhao Y.-M., Zhou J.-M., Wang L.-R. (2012). HIWI is associated with prognosis in patients with hepatocellular carcinoma after curative resection. *Cancer*.

[19] Livak K. J., Schmittgen T. D. (2001). Analysis of relative gene expression data using real-time quantitative PCR and the 2^-ΔΔC^T method. *Methods*.

[20] Cho N.-Y., Kim B.-H., Choi M. (2007). Hypermethylation of CpG island loci and hypomethylation of LINE-1 and Alu repeats in prostate adenocarcinoma and their relationship to clinicopathological features. *Journal of Pathology*.

[21] Eads C. A., Danenberg K. D., Kawakami K. (2000). MethyLight: a high-throughput assay to measure DNA methylation. *Nucleic Acids Research*.

[22] Lin H., Spradling A. C. (1997). A novel group of pumilio mutations affects the asymmetric division of germline stem cells in the *Drosophila* ovary. *Development*.

[23] Ross R. J., Weiner M. M., Lin H. (2014). PIWI proteins and PIWI-interacting RNAs in the soma. *Nature*.

[24] Gu A., Ji G., Shi X. (2010). Genetic variants in Piwi-interacting RNA pathway genes confer susceptibility to spermatogenic failure in a Chinese population. *Human Reproduction*.

[25] Liu X., Sun Y., Guo J. (2006). Expression of *hiwi* gene in human gastric cancer was associated with proliferation of cancer cells. *International Journal of Cancer*.

[26] Liang D., Fang Z., Dong M. (2012). Effect of RNA interference-related HiWi gene expression on the proliferation and apoptosis of lung cancer stem cells. *Oncology Letters*.

[27] Xie Y., Yang Y., Ji D., Zhang D., Yao X., Zhang X. (2015). Hiwi downregulation, mediated by shRNA, reduces the proliferation and migration of human hepatocellular carcinoma cells. *Molecular Medicine Reports*.

[28] Liu W., Gao Q., Chen K. (2014). *Hiwi* facilitates chemoresistance as a cancer stem cell marker in cervical cancer. *Oncology Reports*.

[29] Litwin M., Dubis J., Arczynska K. (2015). Correlation of HIWI and HILI expression with cancer stem cell markers in colorectal cancer. *Anticancer Research*.

[30] Taubert H., Greither T., Kaushal D. (2007). Expression of the stem cell self-renewal gene Hiwi and risk of tumour-related death in patients with soft-tissue sarcoma. *Oncogene*.

[31] Aravin A. A., Sachidanandam R., Bourc'his D. (2008). A piRNA pathway primed by individual transposons is linked to de novo DNA methylation in mice. *Molecular Cell*.

[32] Carmell M. A., Girard A., van de Kant H. J. G. (2007). MIWI2 is essential for spermatogenesis and repression of transposons in the mouse male germline. *Developmental Cell*.

